# Supplementary material for: Opening the black box toward a modular approach to spike sorting
Source: eLife. 2026 Sep 25;15:RP110588. doi: 10.7554/eLife.110588 (PMC13614784; doi:10.7554/eLife.110588)
Supplement: Peer review history

# Editor report: eLife Assessment

**DOI:** 10.7554/eLife.110588.3.sa0

**Authors:** Adrien Peyrache

**Affiliations:** McGill University Canada

**Keywords:** Solid, Valuable

This **valuable** study presents a promising framework for automated spike sorting in high-density extracellular electrophysiology. The core evidence is **solid**, but could be strengthened with extended simulations, broader benchmarking against existing sorters, and validation via expert curation. It will be of interest to the systems neuroscience community.

# Referee report: Reviewer #1 (Public review)

**DOI:** 10.7554/eLife.110588.3.sa1

Summary:

This work presents a flexible spike-sorting framework that allows users to run, swap, and benchmark individual modules commonly used in spike sorting. The paper argues that "opening the black box" is essential for understanding which components drive performance differences and for making progress toward more accurate and transparent spike sorting.

Using this modular benchmarking pipeline, the work identifies electrode drift as a primary bottleneck for accurate sorting, and introduces an end-to-end sorter ("Lupin") that combines the best-performing modules and is reported to be on par or maybe even outperform existing spike-sorting packages on their benchmark. While the modules forming Lupin were chosen based on a single benchmark, end-to-end evaluation of different sorters now use multiple benchmarks, including different available datasets.

Overall, this is a strong tool/resource contribution with clear potential to accelerate spike-sorting development and enable more rigorous comparisons. While not the main point of the paper and therefore less important, remaining claims regarding Lupin outperforming other sorters are not well supported. Lupin does not necessarily outperform all other sorters in real data if you consider 'finding most good units' more important than decreasing false positives - something that quality metrics post-sorting can take care off.

Strengths:

This work has high community value and practical utility. The effort to make benchmarking and spike sorting modules accessible and standardized is substantial, and likely to be broadly useful.

Treating spike sorting as a set of interchangeable modules is a useful approach to some extent, and it enables targeted improvements rather than 'new sorters' popping up which are difficult to fully understand.

Implementing this resource within SpikeInterface, an already widely used tool, will facility uptake and community contributions.

Overall, I am positive about this manuscript as a resource paper. The core framework is compelling and timely.

Weaknesses:

(1) I appreciate the use of automatic curation tools to define the quality of units obtained from different spike sorters. However, a discussion on what is considered a good trade-off is missing. For example, one could argue that as long as you apply these curation tools post-sorting, finding more good-quality units is more important than keeping the number of false positives low, as these can be filtered out at a later stage by these curation methods. The manuscript currently seems to argue the balance between high number of good and low number of false positives is more important. This needs to be discussed and rationalized more explicitly, rather than using terms like 'clearly outperforms' and 'good trade-off'.

(2) Although I agree that overfitting to specific data is a general problem with spike sorting (as you can also change parameters of individual sorters), and perhaps this is even required for the best results, I still miss an explicit discussion of this.

(3) While the end-to-end evaluation is a very good addition, defining the best strategy for each module (which in turn led to choices what to use for Lupin) is still based on one benchmark only. This remains a weakness.

Cmments on revised version:

Previously identified weaknesses in limited support for Lupin being superior to other spike sorters, especially using only a single benchmark, have been largely addressed by (1) making superiority of Lupin less of a point in the manuscript and (2) adding more simulations and real datasets. Also, clarification on serial versus iterative spike sorting has been added to the discussion.

# Referee report: Reviewer #2 (Public review)

**DOI:** 10.7554/eLife.110588.3.sa2

Summary

Spike sorting, that is, assigning events detected in extracellular electrophysiology data to firing of individual neurons, is an inherently difficult computational problem involving multiple steps. The difficulty arises from low signal to noise, instability in signal due to relative motion of the tissue and recording sites, and large volumes of data. Experimental ground truth data - where the correct assignment of spikes in known - is not available in large enough quantities to test algorithms. This paper describes a tool for creating fully synthetic ground truth data and benchmarking the individual steps of spike sorting to dissect the impact of signal to noise, firing rate, and motion correction on each step. This information is used to construct an optimized algorithm for sorting these ground truth data. One result of particular interest is the dominant role of motion correction in degrading accuracy. Another important technical result is that motion correction via interpolation of the voltages traces yields similar accuracy to interpolation of the spike templates.

Strengths

The paper shows that useful insight can be gained through analyzing process step by step. While this analysis has also been done in papers presenting spike sorters (for example, Pachitariu (2024)) the tools presented here allow users and developers to do similar studies for their own work. This toolset will be useful to many labs, especially those working in less studied brain areas or model systems, cases where the tuning of standard spike sorting tools is not a good match to the data.

Weaknesses/Limitations:

The model ground truth data used in testing spike sorting and its components does not need to be a perfect match to experimental data to provide useful benchmarking. However, as with all measurements of spike sorting accuracy, extrapolation to experimental data can be complicated. Therefore, the insights gained concerning optimization of the individual steps should be interpreted as "correct for that model data. The comparison of the paper's new sorter to standard sorters on experimental recordings suggests that the benchmarking data is reasonable. Nevertheless, users of these tools will need to assess how well the simulated data matches their recordings.

# Referee report: Reviewer #3 (Public review)

**DOI:** 10.7554/eLife.110588.3.sa3

Summary:

In this manuscript, the authors describe two additions to an existing toolbox (SpikeInterface, Buccino et al., 2020, eLife). The first addition is an empirical simulator for extracellular recordings, in which spikes from predefined templates are added up with Gaussian noise. The second addition involves granting user-level access to intermediate processing steps along spike sorting algorithms. The authors demonstrate the toolbox by evaluating functions (e.g., event detection) or sets of functions (e.g., feature extraction + clustering) on their simulated data and suggest that a specific combination of function implementations provides performance improvement relative to kilosort4 (Pachitariu et al., 2024, Nature Methods).

The validity of the work is poor. In particular, the simulator is unrealistic and the ground truth dataset is too short. Several spike sorters are used as straw men, and most sorters compared have never been described in peer-reviewed literature or in sufficient detail. The purely feedforward architecture of the modules is very limiting and irrelevant for modern sorters. Finally, the reporting of results is sporadic and does not follow scientific reporting standards.

General comments:

(1) Abstract, lines 14-16: "We then leverage these results to create a modular component-based spike sorter that can outperform Kilosort 4 on dense and large simulated recordings and produce similar quantitative results on real data." However, the artificial data are not "large" - they are very short. And "similar quantitative results" cannot be assessed on real data because those data do not have any ground truth. Because this is a revision and the authors have already received similar feedback from this Reviewer, I am not sure what to recommend.

(2) In a previous comment, I indicated that the simulator itself is overly simplistic, and indicated that as far as I am concerned, the authors must improve it in one of two ways: (1) use a set of biophysical equations, with multi-compartmental modeling of currents and return currents; (2) use noised data from extracellular recordings; or (3) some combination thereof. The authors explained in their answer - but not in the MS - some of the shortcomings of biophysical simulators, but chose to do neither. My comment therefore remains unaddressed in the MS.

(3) In a previous comment, I indicated that the duration of 10 minutes is too short. The authors chose to extend the duration to 30 minutes, which is insufficient for units that have low firing rates. Units that fire e.g., 0.1 spikes/s would have fewer than 200 spikes. If this MS is to be taken seriously, the simulation should be done on durations that (1) are similar to the potential applications - which may be many hours or even days; (2) allow identification of low-firing neurons. Therefore, about 3 hours is the bare minimum. Therefore, at present, my comment remains unaddressed.

(4) In a previous comment, I indicated that some sorters have never been described in peer reviewed papers and therefore, they must either be removed from the present comparisons or be described in full. The authors chose to persist in relying on un-reviewed online documentation. Thus, my comment remains unaddressed, and the comparisons that involve those sorters (e.g., TDC, TDC2, SpyKING Circus 2) are simply invalid.

(5) In a previous comment, I indicated that some sorters (SpyKING Circus 2 and TDC2) are effectively straw men and suggested to reorganize the MS to demonstrate its main goal. Specifically, I suggested to reorganize the manuscript so that after every module is evaluated separately based on a limited ground truth dataset, a single "best" sorter would be constructed, and then tested extensively (and compared to the de facto state of the art). Such reorganization would both demonstrate the utility of a modular approach and clarify the general usefulness of the outcome. The authors agreed that these are straw men and gave a historical account of why these were included. However, they did not explain these considerations in the MS itself, and chose not to reorganize the MS. Therefore, my comment remains unaddressed.

(6) In a previous comment, I commented about the presentation, description, and interpretation of the results, and indicated that the choice to report point estimates makes any conclusions based on those results invalid. The authors did not make any changes to the reporting. Therefore, none of the results reported in the MS can be taken as an outcome of scientific inquiry. In other words, the MS does not deliver any solid findings - neither scientific nor methodological.

# Author comment: Author response

**DOI:** 10.7554/eLife.110588.3.sa4

**Authors:** Samuel Garcia, Chris Halcrow, Charlie Windolf, Zachary M McKenzie, Paul Adkisson-Floro, Heberto Ramon Mayorquin, Benjamin K Dichter, Alessio Paolo Buccino, Pierre Yger

**Affiliations:** Centre National de la Recherche Scientifique Lyon France; University of Edinburgh Edinburgh United Kingdom; Columbia University New York United States; Harward Cambridge United States; CatalystNeuro Casper United States; CatalystNeuro Casper United States; CatalystNeuro Benecia United States; Allen Institute for Neural Dynamics Seattle United States; Lille Neurosciences and Cognition Paris France

The following is the authors’ response to the original reviews.

As requested by all three reviewers we have added a new figure which applies our new end-to-end sorters on real openly available data. This demonstrates that our modular algorithms, optimized to work on simulated data, also performs well in real world cases. In addition, to demonstrate that our results are not due overfitting on simulated data on a specific probe geometry (Neuropixels 1.0), we added a Supplementary Figure demonstrating that our positive results for the end-to-end spike sorters are observed for numerous probe geometries (Neuropixels 2.0, SiNAPS, tetrode and Cambridge NeuroTech). We hope that the extended applications will convince the reviewers and readers of the robustness of our results.

**Reviewer #1 (Public review):**

Weaknesses:

The reviewer identifies several weaknesses:

(1) The main concern is the limited support for the claim that ’Lupin’ and individual modules’ outperform existing spike sorters.

(2) Evidence is primarily from a single benchmark based on an intentionally simplified simulation. While the authors discuss the trade-offs between simulated and real data, the current evaluation does not provide enough diversity to justify claims of superiority.

(3) While improving individual modules that run in a serial fashion could aid overall spike sorting performance, acknowledging that some end-to-end sorters work in an iterative fashion across multiple of these modules would be fair. Perhaps the optimal spike sorter is not a serial set of modules.

(4) There is also a risk of benchmark overfitting. A modular approach makes it easy to select components that excel on specific benchmarks (or a specific project’s data characteristics) without generalizing.

We would like to thank the reviewer for the comments and the valuable feedback. We revised our paper to answer the major concerns that were raised by the reviewer. Regarding the claims about Lupin (1,2), we modified the manuscript in two directions: (i) we attempted to stress that the goal of the paper is not the introduction of the new Lupin sorter *per se*, but rather presenting and highlighting the modularity of the sorting components framework. In doing so, we also toned down our claims of superiority; (ii) we added simulations on a diverse range of probes and included three real experimental datasets, obtained with different probes, in the results. Regarding the iterative aspect of some sorters – point (3) – we added a paragraph to the discussion highlighting that Kilosort4, unlike previous versions of KiloSort, [9] does not iterate over modules anymore, and thus it can be regarded as a serial algorithm. In addition, although all sorters we present are serial, our proposed framework does not prevent iterative schemes: for example, one could add a re-clustering step after a first template-matching pass. In fact, the modular framework makes this task even easier than before.

Finally, related to point (4), we added some comments on the problem of overfitting with modular benchmarks in the Discussion, but we think that the risks have been mitigated with the addition of more end-to-end examples with various probe types and experimental data in the revised manuscript.

The reviewer also points toward some possible ways to strengthen this work:

(1) Evaluate on multiple simulation regimes, consider adding at least one biophysically detailed simulation, benchmark on multiple probe-geometries with neurons also clustered in different depth profiles (as this will affect drift solutions), and provide real-data validation. Even without full ground truth, real-data can be evaluated with expert curation, functional validation (e.g., refractory violations, quality metrics, unit waveform consistency), agreement across sorters, and consistency across time.

(2) Related to real-data applicability, it is also important to acknowledge that modulatory approaches can enable overfitting to the needs of individual projects. Without real-data benchmarking (or benchmark diversity), it is unclear how the framework will guide users towards generalizable ’best practices’ rather than optimized configurations that work for their specific conditions.

In response to these comments, the manuscript has been extended both with more simulated ground truth recordings and experimental data. For additional ground truth, we generated recordings with various probe geometries, to check that the results observed for our modular pipeline could generalize (see Supplementary Figure). Regarding real experiments, we chose three open dataset of various types (chronic and acute implantations, IMEC and Cambridge Neurotech devices) and compared the results of several sorters at a macroscopic level relying on high-level automatic curation tools [7, 3, 6] to quantify how many “good”, “oversplit” and “noise” units are found. This is now a new Figure 8 in our manuscript. We believe that the results from all these datasets demonstrate that Lupin is, as is claimed in the paper, *on par* with the most popular spike sorting algorithm, Kilosort4 [9].

Regarding overfitting, we do not believe this is a direct consequence of the modular approach introduced in this article, but rather a general potential “risk” in the spike sorting field. Each spike sorter exposes a large array of parameters that users can tweak to attempt to optimize outcomes on specific datasets, but this end-to-end fine-tuning is hard to control and quantify. We believe that the modular benchmarks introduced in this paper may enable a finer, more controlled, and quantifiable parameter exploration. As an example, very high firing rates such as those observed in the cerebellum might require different parameters for peak detection than for neurons in the cortex. To test this, one could use our generation framework to mimic key macroscopic features of the system you are studying, and benchmark the peak detection step to find optimal parameters. Overall, we do not see this optimization strategy as a problem. Extracellular electrophysiology is so diverse based on brain regions, species, conditions, tasks, etc. that generalizable “best practices” might not exist.

**Reviewer #1 (Recommendations for the authors):**

(1) Tone down or further support the Lupin and specific modules’ superiority claims.

This has been modified in the manuscript, and we added a final figure to discuss how Lupin is *on par* with Kilosort on real data, but with no claim of superiority.

(2) Add benchmark diversity, this would test generalization and mitigate benchmark overfitting. Specifically: add more probe-geometries, and allow for different depth-profiles of clusters of neurons.

We thank the reviewer for the suggestion, and indeed, we added some more benchmarks to convince the readers that the results observed can be generalized properly. More specifically, we extended the duration of the recordings to 30 min, and included additional benchmark datasets with four different probe geometries (Neuropixels 2.0, a Cambridge Neurotech probe, a SiNAPS probe and a tetrode).

(3) Clarify how (x,y) positions of neurons are distributed around the shanks.

This has been clarified in the Methods section. The (*x,y*) positions are generated uniformly within a rectangle covering the probe boundaries, plus a 20µm margin. Regarding the depth, *z* positions (distance from the probe) are drawn uniformly from the range [5,40] µm.

(4) Add at least one real-data benchmark. Some suggestions for evaluation are: stability of firing rates over time, agreement across sorters, quality metrics, functional validation, expert curation.

As suggested by almost all reviewers, we added some real-data benchmarks (see last figure). Since experimental data don’t have ground truth, we used automatic curation tools as a proxy for “goodness” of the results. We used automatic labels from Bombcell [3] and UnitRefine, which label units as good, multi-unit activity (MUA) and noise, and SLAy [7] for automatic merging, which correlates with the amount of putative oversplits. We felt it was fairer to use external curation tools instead of creating our own methods to assess quality.

(5) Clarify recommendations for users facing drift. While your statement of ’not having drift is ideal’ is true, the reality is that many recordings have drift. Some practical solutions would be useful. For example, when to trust results and how to report drift sensitivity.

The reviewer is right, and we added a sentence to clarify when motion correction methods should be used, in our opinions.

(6) It would help to see what types of signals are most often missed, for example: low-SNR units, drifting units, bursty units, and show which modules affect which of these issues.

This is already shown in Figures 4 of the manuscript, at the clustering level. These Figures show that cells with low firing rates and/or low SNR are most likely to be missed by all sorters. Our ground truth simulator does not include a bursting mechanism yet. We think that this would be an interesting aspect to simulate and we plan to include bursting units, with bursty spike trains and waveform modulation, in future releases. We thank the reviewer for the suggestion.

(7) To overcome the issue of overfitting to specific datasets rather than generalization, it would help if a ’default’ or ’recommended starting point’ for users were described in more detail.

We overcome the issue of overfitting by adding other artificial ground truth recordings (see Supplementary Figure S1), and also real world dataset (see Figure 8). In all these simulations, Lupin is used with default parameters, and this is, we believe, a good starting point. Of course, for very special needs (animal species, brain structures, ...), one might need to adapt parameters, but so as for any other sorters, and such an exploration of the parameter space is out of the scope of the current manuscript. This has been added in the Discussion.

(8) A lot of the plots have ticks / labels too small to read in 100%, or show quite low-resolution. For example, Figure 3 and Figure 5. Please homogenize across all figures.

The figures have been regenerated and homogenized as suggested.

(9) Consider archiving the GitHub version used to generate the figures on Zenodo (DOI) for posterity.

This has been done for the current state of the manuscript at https://zenodo.org/records/20407862 and the code is available at https://github.com/SpikeInterface/sorting_components_benchmark_paper

(10) To make the manuscript more reader-friendly, I recommend adding graphics representing the different methods. For example, in Figure 3 one could add schematics of the two peak-detection methods.

We thank the reviewer for this suggestion. However, this project and our manuscript is not introducing these methods, only re-implementing them in a modular framework. Hence we feel it is out of the scope of this manuscript to produce schematics of the many methods discussed in the paper readers should view the original sources to find out more information.

(11) Discuss what is meant by KS-like clustering, as I was under the impression that KS4 is also iterative. This may be on the template-matching side, but it’s difficult to know where you draw the border between iterative clustering and (iterative) template-matching. Potentially, we would want to see these processes as one module together, as many sorters work in an iterative fashion across these steps.

By KS-like clustering, we meant that the code is a direct port, in Python, of the clustering algorithm implemented in KiloSort4. However, we cannot guarantee that this is the exact same clustering method, because of the way the clustering of KiloSort is interleaved with some others steps part of the algorithm. To be more specific, Kilosort has its own special way of performing matched filtering, with a custom grid of templates generated internally with a higher resolution compared to the recording channels positions. These templates are then used to estimate a putative position of each spikes, and these positions are the ones that are used to start the clustering. Thus, the term KS-like comes from the fact that we do not reproduce this exact same mechanism. In SpikeInterface matched filtering is performed using an equivalent approach, but positions are not estimated in the exact same manner. Since the clustering code is equivalent, the results might differ. Regardless of these details, the clustering is not iterative. In former versions of KiloSort, the core algorithm used ideas from k-SVD algorithms, often used in the Machine Learning community. In such an algorithm, the goal is to learn a sparse dictionary of templates to reconstruct the signals, and indeed, there was some iterations between optimizing the templates and the spike times. However, this is no longer the case in KiloSort4. The algorithm works in a serial manner, following the global strategy mentioned in our paper.

(12) Rather than showing results for one specific simulation, it would be more convincing if we saw the average result of multiple simulations. We don’t need to see individual neurons necessarily (e.g., Figure 3B and C, but this applies throughout the manuscript).

While we tend to agree with the reviewer than averaging over multiple datasets might be more informative (as we did in the final figure, for end-to-end sorter comparison), we want to say that given the fact that we are using ground-truth recordings that are randomly generated, as long as we do not change the macroscopic properties of the recordings, results on various instances of the noise will be very similar, and averages might not be as informative as one could hope for. One option would be to vary the parameter space of these ground truth recordings, but then there are so many parameters (noise levels, firing rates, distributions of the cells, ...) than averaging everything, and/or even choosing what should be primarily studied is an open question on its own. However, to ease the redibility of the figures, individual neurons were removed from the plots.

(13) Legends are often incomplete. For example, describe what individual dots are and what lines are in the different figures, even if it seems obvious.

Legends have been updated.

(14) Figure 7 would benefit from average thick curves for each model (optionally with individual thin lines for each instance, or error bars). Individual neurons can be left out. Figure 7E (and likewise Figure 5E) would benefit from having x labels to indicate precise labels, so the color attribution is reserved for specific models. It’s quite an intense ’search’ game to understand these figures.

All the figures have been regenerated, and for the sake of readability, we removed the scatter plots for the individual neurons, focusing only on the averaged lines.

(15) While I appreciate the effort is gigantic, it may be better for the reader to conclude that.

This has been rephrased.

**Reviewer #2 (Public review):**

Major comments

The model ground truth data used in the paper does not need to be a perfect match to experimental data to provide useful benchmarking. However, as with all measurements of spike sorting accuracy, extrapolation to experimental data can be complicated. Users of these tools will need to assess how well the simulated data matches their recordings.

We agree with the reviewer that extrapolating our results to real data is difficult, and the same point was raised by the other referees. We have now extended the results to include three experimental datasets from different probes. Due to the lack of ground truth, we used automatic curation tools (Bombcell [3] and UnitRefine for labeling, SLAy for merging [7]) as a proxy for performance and showed that Lupin is on par with Kilosort4 on all datasets. We hope this gives users some idea of how well the new sorters will work on their data.

**Reviewer #2 (Recommendations for the authors):**

(1) Any comparison to experimental data would be welcome. Is it possible to add firing rate, amplitude, and template similarity distributions from measured recordings to the panels in Figure 2D?

Experimental data is very diverse, depending on brain region, species, recording technology, etc. Instead of extending the comparison between simulated and experimental data, we rely on new Figure 8 to showcase the applicability and performance of the presented methods on real recordings.

(2) Do yields of units passing basic quality metrics look different with the new sorter vs. established sorters? Another option that would help establish the range of applicability of the results would be a different model ground truth system, e.g., the hybrid ground truth data used by the authors in reference 7.

As it can be seen on synthetic recordings (e.g Figure 7A-B), all sorters behave similarly with respect to firing rates, signal to noise ratios. To help establish the range of applicability, we added an extra Figure 8 in the manuscript, as described above.

(3) Interpolation errors are likely larger for NP1.0 probes - which have 40 um vertical pitch - than NP2.0 probes, with 15 um pitch. If it’s possible to include even a small-scale comparison of the results from the 2.0 geometry, that would be very valuable for readers trying to decide what probe type to use.

In the revised manuscript, we added new ground truth benchmarks with a NP2, and other, layouts to show that the key results do not depend on the geometry of the probe (see Supplementary Figure 1). But to address more specifically the point raised by the reviewer, we note that in the case of applying Lupin to NP2 layouts, there is only a loss of ≈ 7.5% of well-detected units between static and motion-corrected cases (see Supplementary Figure 1). Where when we apply Lupin to NP1 layouts (see Figure 7) there is a decrease of 20% of well-detected units. So clearly, a smaller pitch and the columnar arrangement of NP2 seems to help motion-correction methods, and thus reduce the failures due to motion. This has been added in the Discussion.

About the text:

(1) In panel E of Figures 5 and 7: Adding the name of the sorter algorithm under the bar charts would be helpful. It is encoded by the color of the outline of each box, but I found that cue rather subtle.

The figures have been regenerated, with increased ticks and label fonts. We found that adding the names made the Figures too dense, and acronyms would not simplify the figures. In the end we decided not to add them.

(2) Is the number of features (K) used for clustering already mentioned in the main text or methods? I couldn’t find it.

We thanks the reviewer for pointing out this problem, and the answer (*K* = 5) has been added in the manuscript, in the methods section.

(3) Equation 2 in Methods, defining accuracy, appears to be incorrect. I believe the correct equation is: accuracy = TPij/(Ni + Nj - TPij).

The reviewer is right, and this has been corrected

(4) In the abstract, line 18: Component based spike sorters => component based spike sorter.

This has been corrected

(5) Line 169 "we can artificially boost the signal-to-noise..." I don’t really see anything artificial about leveraging the extra information in neighboring sites. Maybe just remove that adjective?

We removed “artificial” from the sentence.

(6) Line 196-197, describing the result in Figure 3: Especially since 3A is a log plot, it would be helpful to add a percentage to the spikes missed on the high end. These are probably pretty unusual cases, under 0.1%?

This has been commented in the text, but both because this number is only an approximation (the problem of pairing peaks between detected and ground truth is slightly ill-defined), and because it depends on the particular seeds and parameters of the artificial ground-truth, we avoided numerical values.

(7) Line 567: "number of dimensions from M to K 5" => "number of dimensions from M to K[5]" That is, is the 5 meant to be a reference? Or is 5 the number of dimensions retained to describe the temporal waveform?

5 is the value of K, and this has been corrected in the manuscript.

(8) Line 585-590: Does this description of how to handle borders between groups of sites also apply to the KS-clustering method?

For the KS-clustering methods, we used the original method implemented in KiloSort. In fact, KiloSort aggregates all the clusters found during the clustering steps, launched per bins, but duplicated templates (based on their shapes only) are removed before matching (and those are the cells at the borders found numerous times). After the template-matching step, once cells have been “populated” with theirs spikes, templates are once again removed and/or merged. However, as explained in the methods, currently this step has not been implemented in our framework. To be more explicit, during template matching, KiloSort stores the features of all discovered spikes, in a space where the effects of nearby spikes have been subtracted, to get a clearer picture of these features. In this “denoised” space, the clustering algorithms is launched again on all spikes (decimated), to assign labels.

(9) Line 687: "In fact, a major main with Kilosort lies after the template matching step..." => "In fact, a major difference with Kilosort lies after the template matching step...".

This has been corrected.

**Reviewer #3 (Public review):**

Major comments

(1) The simulator itself has to be improved and extended. Right now, it simply generates, for every unit, a mother waveform from a sum of exponentials, scales that over channels, and then adds up multiple instantiations of every unit on every channel, along with noise. This is not a biophysical simulator: it is an ad hoc procedure, and the sentence "we firmly believe that.." (lines 482-483) does not make the procedure convincing. To make the simulator credible, the authors should: (1) use a set of biophysical equations, with multi-compartmental modeling of currents and return currents; (2) use noised data from extracellular recordings; or (3) some combination thereof.

The reviewer is right when pointing out that the current ground truth generator is not “biophysical”, and this is why in the manuscript we used the terms “biophysically plausible”. However, we decided to changed this phrase to “phenomenological” in order to avoid confusion. We believe that our generation tool has the key ingredients to challenge (and also demonstrate limits of) modern spike sorters, which is the goal of the proposed simulator. Spike sorters performing well on such phenomenological simulated data should be a necessary, but not sufficient, condition to convince experimentalists that they will work well on real data. In previous papers [5, 4], we used MEArec [1], which relies on biophysical modeling of the reconstructed neurons to simulate extracellular potentials to generate ground-truth recordings. However, such biophysical simulations have two shortcomings: (i) simulations are very slow and resource-hungry; (ii) it is not guaranteed that the superior simulation environment (multicompartment modeling) translates to simulated data that are more similar to experimental data. In fact, the Kilosort4 paper [9] shows that the action potentials generated by MEArec [1] have an almost doubled duration compared to realistic data, which requires further ad-hoc parametrization. We believe this discrepancy can be due to the fact that virtually all multi-compartment models are built from in vitro slices, not in vivo recordings. Given these limitations, we decided to rely on a simpler but better controllable model for generating templates. Despite not being “biophysical”, the generation model can replicate, to some extent, the variability in waveforms (using different parameters for waveforms widths and spatial decays) and allows us to have a ground-truth model for drifting as well, that we can use to assess interpolation errors. Nevertheless, we agree with the reviewer that some aspects of the simulator can be improved, such as the structure of the noise in the data. We are currently working on the Spikeinterface side to improve the generation module so that it supports temporally correlated noise (spatial correlation is already supported and used in this manuscript).

(2) The simulated dataset has to be extended in time. Maybe I missed something, but 500 units over 10 minutes, with some units having firing rates as low as 0.1 spikes/s, corresponds to some of the units firing an expected 60 spikes. This is clearly too short, and does not replicate the standard situation in extracellular experiments.

We extended the simulated recording to an half an hour duration. However, as it can be seen in the Figures, this does not affect the main results of the paper.

(3) The simulated dataset has to be extended in space. The choice of using NeuroPixels 1.0 geometry is a poor one. Many labs use other monolithic electrode arrays (MEAs, silicon probes, other rigid arrays); tetrodes remain a major tool, and flexible probes (polyimide, mesh) are evolving. Assessing algorithms over a single spatial architecture is likely to lead to local maxima in performance and potentially erroneous conclusions.

We believe the that Neuropixels 1.0 geometry is a good choice: the NP1.0 paper it is the most highly cited paper about a high-density electrophysiology probe, suggested that it is currently the most widely used probe in the world. However, the reviewer is correct that there is a risk of overfitting. To demonstrate generalizability of our algorithm, we have added results obtained with NP2.0 layout, a Cambridge NeuroTech layout, a SINAPs probe layout and tetrodes. The results demonstrate that the Lupin sorter is not overfitted.

(4) The existing spike sorters evaluated are not completely described. Some sorters (e.g., SpyKING Circus and KS4) were described in previous publications, but it is unclear whether the implementation that was used for the present tests is exactly the same as those previously published. More importantly, some of the sorters evaluated (e.g., TDC, TDC2, SpyKING Circus 2) were never described in a peerreviewed paper. This does not mean that they cannot be evaluated - but if they are, they must be described in full. Relying on the fact that the code is open source cannot replace a complete and accurate scientific description.

The reviewer is rising a valid point, but we think it is beyond the scope of this paper to fully describe every component of every spike sorter mentioned in the paper. We made the deliberate choice to describe the sorters as chains of components in order to demonstrate the flexibility of our modular approach. Full details of every components can be found in the online documentation. In order to ensure the manuscript felt more complete and concrete, we revised the descriptions in our Methods section, in order to give some more details.

(5) Related to the above, all relevant code should be made available online in permanent repositories, not only in author-controlled ones.

We are not sure of what exactly is suggested by the reviewer. In order to clarify the situation, we pushed the notebooks and all the code needed to reproduce the Figures of the paper in a Zenodo archive https://zenodo.org/records/19695406

(6) It is unclear why SpyKING Circus 2 and TDC2 are evaluated - these could potentially be described as straw men. I recommend reorganizing the manuscript so that after every module is evaluated separately based on a limited ground truth dataset, a single "best" sorter would be constructed, and then tested extensively (and compared to the de facto state of the art). Such reorganization would both demonstrate the utility of a modular approach and clarify the general usefulness of the outcome.

Although we agree that these two sorters might be seen as straw men, we decided to keep them in the paper for various reasons. This has been clarified in the manuscript, but the primary reason is an historical one: the developers of these sorters decided to unite their efforts while designing new tools and algorithms, which led to the initial work on the modular framework described here. Because SpyKING CIRCUS and TriDesClous had to evolve for maintenance, it was decided to try to write a common “grammar” that would allow these two spike sorters to be described in the same framework. The reviewer is right in the fact that once the foundations were stabilized, most of the development efforts were put to Lupin, that was built as the best combination of all the expertise gained on the two aforementioned sorters. A second reason to keep them is that, once again, we decided that Lupin should not be the main focus of the paper. Of course, this is a nice illustration of what the modular framework can do, but we do not want to push it *per se*. What matters most is the methodology, especially since we can not claim, here, that Lupin would be the best spike sorter regardless of data types, probe geometries, .... We extended the paper with other probe geometries (see added Supplementary Figure 1) and real world data (see Figure 8), and we observed that Lupin was on par, and/or slightly better than KiloSort 4 with respect to number of False Positives for examples. But ultimately, the paper is really about the development of a common ecosystem such that all tools can be improved upon, at the community level.

(7) The new algorithms developed, for example, clustering and template matching, have to be described in more detail, and demonstrated graphically on simple datasets. This can be done in supplementary material if the authors prefer not to extend the manuscript too much.

As suggest by the reviewer, we tried to extend the methods of the clustering and the template matching steps, bearing in mind that some of them have already been published in detail elsewhere. We really want to underline that the central point of the paper is the modularity of the architecture, not so much the low-level details. To populate our framework and demonstrate its generalization, we implemented some key algorithms. But describing with schematics and in depth every individual methods is something that is not even done in papers focused on the spike sorters themselves. Later, the reviewer complains that the paper sounds like a technical report. Delving into more details would only amplify this problem.

(8) This reviewer finds the description and interpretation of the results to be inadequate. As an example, focusing on Figure 5: The results in Figure 5A have to be supplemented and summarized as a scalar point estimate (e.g., median accuracy), an estimate of dispersion (e.g., using MAD, IQR, or SD), evaluated over multiple runs, and compared using statistical tests between tools and conditions (e.g., using a multi-dimensional analysis of variance, a mixed effect model, etc.). The results in Figure 5D must have an indication of dispersion. Any conclusions based on the numerical experiments must be based on these metrics and statistical evaluations.

To try and simply the plots and their interpretation, we decided to remove the scatter plots of the individual neurons in all Figures, to really focus on the core trends. We believe that the results, such as the dependence on accuracy as a function of SNR, are difficult to capture with summary statistics, and that the results are best understood by looking at the plots we have made. If we wanted to make strong claims about one algorithm being more suitable for a specific task, then these summary statistics would be suitable. Instead, we are trying to demonstrate the general utility of the components framework, and we optimize Lupin simply to maximize the accuracy of each step.

(9) The entire MS would benefit from expert proofreading; there are many language errors, mostly in indefinite articles and grammatical numbers.

The manuscript has been intensively proofread by native english speakers.

**Reviewer #3 (Recommendations for the authors):**

(1) Lines 14-15: "...a... sorters...": either "...sorter..." or "...a... sorter...".

This has been corrected

(2) Line 30: "peak detection" or "event detection"?

We prefer the term “peak detection", since this is exactly what the algorithm are looking for: spatio-temporal extrema in the signals

(3) Line 30: the purely sequential structure of the modules is very limiting and generally incorrect. Template matching may replace peak detection, as is the case in many real-time hardware implementations. The feedforward process is limiting, and many sorters use feedback or multiple loops. It is unclear whether and how the proposed framework supports such structures.

The reviewer raises a good point that we did not explain well in the manuscript. Since our framework is modular, with each component independent of the others, a developer has freedom to create “iterative" sorters. E.g. they could loop over a pair of steps until a criteria is met. In fact, this is one of the advantages of creating modular components. The examples we show in the manuscript are sequential feedforward structures, leading to this confusion. We have clarified the point in the text. We show sequential sorters because to our knowledge there are currently no iterative sorters in wide use. Older versions of Kilosort were iterative, but KiloSort4 is not. Modularity also allows us to isolate one component for a specific task. Hence, for a real-time implementation, we can pre-compute templates using the peak-detection and clustering components. Then these steps would be skipped for “online” sorting, which would only use the template matching component. The reviewer states that “template matching may replace peak detection”. Indeed, in Lupin, SC2 and TDC2, template matching does replace peak detection – the initial peak detection is only used to construct templates for downstream matching. We have clarified this point in the text.

(4) Lines 62-63: Are all of these sorters supported by the spike Interface framework? Please include a table of which are and which are not. The same for every module.

All the sorters listed are indeed supported by Spike Interface, and this has been added in the text.

(5) Lines 84, 98, and elsewhere: TriDesClous 2 is mentioned. What about TriDesClous - is there such a sorter, and if yes, what is the scientific reference?

TriDesClous (https://github.com/tridesclous/tridesclous) is a spike sorting pipeline that has not been properly published with a DOI, but that has been developed by the first author of the manuscript and has been used by many papers [8].

(6) Line 84: Lupin - suggest reorganizing the manuscript around this sorter and evaluating it on multiple datasets.

The paper is not about Lupin, and we tried to rewrite the manuscript in order to make this point more explicit. The paper is intended to be a proof of concept of the benefits that can be obtained thanks to a modular approach. This is why we do not want to reorganize the paper on Lupin itself.

(7) Line 104, Figure 1, and elsewhere: What is the advantage of evaluating three sorters, if two are predicted to be worse than the third/state of the art? Suggest to reorganize the MS: (a) describe a proper simulator; (b) describe every individual modules: mention the existing algorithms and elaborate + demonstrate the new algorithms; (c) evaluate every individual module - on properly realistic dataset; (d) evaluate existing complete spike sorters + the proposed best combination - on the same ground truth dataset; (e) compare the best two sorters on multiple datasets. In addition, may identify the WEAKEST link in each sorter and demonstrate the improvement by replacing ("upgrading") that link alone.

As clarified in the introduction and in the “End-to-end evaluation..." section, we decided to keep three sorters for various reasons. The first is historical: SpyKING CIRCUS 2 and TridesClous 2 were the first two sorters that motivated the creation of the sortingcomponents framework. Because both authors realized that they had so much code in common, they decided to unite their efforts while rewriting them and share some common building blocks. The second reason is that the paper is not about Lupin, *per se*, but more about the general philosophy of the modular architecture presented here. We want to push forward the idea, in the community, that we should share tools, ideas and algorithms in order to enhance the analysis pipelines. Keeping several sorters, even if sub-optimal, is a way to showcase the flexibility of the framework, and this is why we did not re-organize the manuscript as suggested by the reviewer.

(8) Line 121: "can drastically cut the time.." - provide quantitative support. In general, avoid superlatives and unsupported statements.

Spikeinterface has been primarily design to ease the comparison between spike sorting pipelines [2]. Thus all comparison metrics such as agreement matrices, false positives, false negatives, ... are available out of the box when using these Benchmark objects. It would be hard to provide a quantitative support for such a speedup since it will depend on the algorithm, but it allows developer to simply focus on the core implementation while benefiting for free of the whole ecosystem that will launch benchmarks and compare it, with appropriate metrics validated by a large community. Since this validation process, on its own, can be quite complex depending on the processing step, we truly believe the development gain is important, despite the fact that it might be hard to quantify. However, we rewrote the sentence in order to avoid superlatives.

(9) Line 129: "powerful and fast way to generate artificial" - again, avoid statements with superlatives and lacking quantitative support.

We rewrote the sentence to avoid superlatives.

(10) Line 129: "powerful and fast way to generate artificial" - the assumptions made in constructing the artificial data critically and strongly affect the conclusions of the benchmark processes. For instance, it is well known that about 10% of the spikes in the cortex have a positive extrema, but the generator is limited to negative spikes. Also, the generator produces spatially-displaced and scaled versions of the waveform generated by the putative soma, but extracellular waveforms almost never behave that way. Even if the simulator is improved, it will always remain a simulator, and therefore the caveat should always be kept in mind.

The reviewer is right: our simulator has some limitations compared to biological data. But the fact is that, even on synthetic data, there is still plenty of room for improvements of current spike sorting pipelines before even getting to real data, where ground truth are unknown. We believe that such ground truth simulator is a necessary, but not sufficient, condition to validate sorting algorithms. Other options such as hybrid recordings would also suffer from the same flaws, and might be even more questionable. In the revised version of the manuscript, we also added tests on real data to compare more qualitatively Lupin and KiloSort. The fact that results are in line with what is observed on synthetic data gives us confidence in our observations.

(11) The authors should add a limitations section to the MS.

Some limits have been more extensively discussed in the Discussion of the paper, with respect to the feedforward architecture, the validity of the ground truth data.

(12) Line 129: Can the benchmark object be used with other data (e.g., existing)? The methods indicate that this is the case - please demonstrate.

We think that a proper demonstration would be out of the scope of this paper, but indeed, as long as the user can provide a recording alongside with a sorting (exhaustive or not), then the Benchmark objects can be used. This allows the use of hybrid recordings, and/or manually curated datasets where users would be able to provide a ground truth. Of course, depending if the ground truth is exhaustive or not (if one knows the activity of all the neurons in the recordings), the metrics might not be the same. But everything is built-in in the object.

(13) Line 144: 10 minutes is too short and nearly irrelevant. In particular, many units spike at rates much lower than 0.1 spikes/s, and in 10 minutes would emit less than a score of spikes (e.g., 0.01 spikes/s would accumulate, on average, 6 spikes..).

We regenerated all the figures in the paper with 30 min long recordings, and the results remain similar to the 10 minute recordings.

(14) Line 151: firing rates are not independent of the waveform as implicitly assumed; this should be accounted for, at least in the options in the simulator. Same for library waveforms - the firing rates should be a parameter that is optionally provided along with the waveform library.

We are not sure what is meant by the reviewer. We believe that the point is that some cell types, with particular waveforms, have particular firing rates, such as fast-spiking interneurons. This could be dealt with in the current simulator, since users can provide, on a per cell basis, some particular values to generate the waveforms of the neurons. One could clearly imagine having some particular cell types with dedicated waveforms and firing rate parameters. However, for the sake of simplicity in the paper, we chose not to use such granularity. What the simulator can not do, at the moment, is to perform amplitude modulation of the templates as function of bursts for example. But this could easily be implemented, and should be part of future works to consolidate the generator.

(15) Figure 2A, D: It is unclear why the specific zig-zag motion was simulated. Please rationalize, or use a motion from a real dataset. In particular, simulate (a) breathing-induced micro-motions, (b) gradual drift, and/or (c) a step jump.

We used a zig-zag plus a Brownian motion to cover a rather broad range of continuous motion. Of course, as pointed out by the reviewer, the heterogeneity of real drifts is large, and again, we believe it is out of the scope of the manuscript to cover them all. In previous works, we explored how motion correction methods were working as function of the drifts [5], and the conclusion was that this simple continuous drift was already challenging enough to make the spike sorters fail. Adding discontinuities, as often encountered in experiment, would only make things worse. Finally, we added real world data (Figure 8) from three randomly picked dataset with heterogeneous probe geometries. They all come with some drift, that might be representative of what is typically dealt with.

(16) Line 181: "more computationally demanding" - quantification?

We forgot to add the reference to panel 3D, and this has been corrected in the manuscript.

(17) Line 192: "(Figure 3A" - add ")"

This has been corrected

(18) Lines 195-6: "... not that missing a few spikes at peak detection will not have a large impact..." - this statement should be quantified.

We reformulated the sentence, but the point here is simply that template-matching based algorithms use the template-matching step exactly for this purpose: to label spikes that would have been ignored/missed by the peak detection and clustering steps. The fact that template-matching based pipelines such as KiloSort, SpyKING-CIRCUS, ... outperforms clustering-based solution when detecting spikes [4] is a clear support for our sentence.

(19) Lines 195-6: "... not that missing a few spikes at peak detection will not have a large impact..." - this statement, if correct, exposes a key weakness of the purely modular approach. For instance, assume that module 2.1 performance can be 0.5 and module 2.2 performance is 0.9, and that will have zero impact on the overall performance of a sorter that has module 4.1 as its fourth module, yielding an overall performance of 0.7. But when module 4.2 is used, module 2.1 performance of 0.5 results in an overall performance of 0.5, whereas module 2.2 performance of 0.9 translates to an overall performance of 0.9. The point should be clear now: evaluating every module in isolation cannot fully predict the behavior of the full system - even if a purely feedforward, single iteration (no loop) architecture is assumed. This requires algorithmic support in the proposed framework, and at the very least explicit discussion.

The reviewer is right, benchmarking every module in isolation cannot fully predict the behavior of the full system. However, the fact that Lupin, built as an optimal combination of the components and can be *on par*, if not better in some situations, than KiloSort 4 on various artificial and real dataset makes a compelling argument in favor of assembling the best algorithmic pieces one after the other. In fully assembled spike sorting pipelines, the failures at one stage might be compensated by some algorithmic optimizations latter on. But still, being able to identify these failures, and eventually correct them at the appropriate level, i.e. as soon as they appear might be beneficial for the development of the tool, and to ease their readability/maintenance. This has been added in the discussion.

(20) Line 203: "we hope that future efforts": This statement is strange for two reasons: (a) if the authors hope for it, why not simply do it? (b) if the authors cannot do it / it is out of scope, the proper place to mention their hopes is in the Discussion section.

This has been moved to the Discussion section

(21) Line 232: "motion is only partially compensated for" - so what is the utility of the compensation? This becomes clear later, but the statement is obscure at this point - please clarify.

This has been clarified.

(22) Figure 4A, right: The fact that the lines do not reach unity even for high FRs suggests that the FRs are NOT the key limiting factor. Please provide a scalar measure and a 2D analysis of the accuracy as a function of FRs and SNRs, separately for static and motion-corrected.

We are not sure that we understand what is being requested by the reviewer. A scalar measure with a 2D analysis of the accuracy as a function of FRs and SNRs would mean, per case (static and motion corrected) at least 4 panels, thus a total of 8 panels. This seems like an overly dense figures. Further, a scalar measure is unlikely to provide more insight into the analysis.

(23) Line 275: "kriging method" - describe.

For a full description we refer readers to the original paper describing the method [9] and other work on motion correction [5]. We have added a brief description of the method in the text in the "Motion interpolation reduces spike sorting performance" section.

(24) Lines 309-310: Where are the templates from in this case - true or estimated? If the latter, say it.

This has been clarified.

(25) Line 317: This is the point where I almost gave up - the MS up to this stage seemed very much like a technical report and is not organized in a clear, results-oriented manner. Even for a Methods-oriented paper, one expects to learn the key results, but those are lost in this MS.

While we agree that this part of the manuscript is rather technical, this is something that we believe is at the core of some scientific questions that have not been yet properly addressed by most of the spike sorting pipelines. The idea of performing template-matching, i.e. to seek for spatio-temporal pattern in the signals while at the same time compensating only partially the motion has never been properly benchmarks. While template-matching is clearly a game-changer in static recordings, i.e. without motion, the extra-addition of motion might limit its performances. We firmly believe that our modular approach can be used to isolate such core questions from the whole integrated pipelines, and guide both users and developers.

(26) Figure 6A, second and third panels: The fourth (rightmost) panel shows that there are differences between the "true" and "interpolated" templates, but this cannot be seen in the two middle panels - probably because too much information is overloaded on every channel. My suggestion is to show a reduced number of channels, but for each of those, show all five waveforms (corresponding to different spatial positions of the centers of mass) in a NON-overlapping display.

The figure has been redrawn, as requested by the reviewer.

(27) Figure 6 and the entire issue of the failure in motion correction + lines 331-332: It is not 100% convincing that the failure is not at the DREDGE level - please demonstrate that the motion is estimated perfectly.

We checked that the DREDGE algorithm is correctly estimating the motion. To convince the reader this is the case, this has now been shown in Figure 2, panel D, where the simulated motion is displayed on top of the motion estimated by DREDGE. As shown, both are very similar.

(28) Figure 6 and the entire issue of the failure in motion correction: If motion is estimated perfectly and interpolation is done properly, then is the failure an outcome of discrete spatial sampling? In other words, if the movement in space over time (in the simulator) were limited to discrete steps that correspond exactly to the positions of the electrodes, would the motion correction allow perfect performance? Stated differently: if the electrodes were not 15 or 20 micro-meters apart but rather 1 micro-meter apart, would the difference (e.g, between Figure 4A/4B, or between Figure 5A and Fig. 5B) be reduced? Please check and report.

The reviewer is raising an interesting point, and indeed, this is something that we are planning to investigate. We felt it would have been too technical for the scope of this paper, which is aimed at showcasing the modular framework and its possibilities. But we are planning to explore such failures with ultra-dense probes as has been done in the DREDGE paper [10]. We also have the intuition that errors are originating from failures of discrete spatial sampling: hence the larger the sampling, the more pronounced the errors.

(29) Figure 6B: It is impossible to discern which line is which - this underscores the general point of summarizing every CDF by a scalar with measures of dispersion (i.e., descriptive statistics) + statistical testing (i.e., quantitative statistics).

While we agree with the reviewer that the plots are dense, they convey the global message of the paper and are the same that were used in [9]. To ease the comparison, we decided to stick to this representation.

(30) Figure 6D: add error bars.

There are no error bars, since there was only a single run in this figure, i.e. on a single dataset.

(31) Line 318, line 328: So what is the result here - that the interpolation idea is not useful? If yes, what is the source of the failure? Is it discrete/poor spatial sampling as suggested above? Something else?

The result is that interpolation, rather than motion estimation, is the bottleneck in recoding with motion. A careful analysis using data from dense probes, as already stated above, would be the focus of further work. But we suspect that errors results mostly from bad interpolation due to discrete spatial sampling.

(32) Line 334: up to this point, there are no quantified results - and actually, no clear results at all.

We rewrote the section accordingly, to make the key observations more striking. The goal of this section is not to propose quantified results and say which interpolation methods is the best (a full paper should be devoted to this question), but rather to show the possibilities offered by the modular framework, looking at questions that have been not yet properly addressed in spike sorting algorithms.

(33) Line 345: "This is likely due" - provide support? Example?

We rephrased the sentence accordingly.

(34) Line 349: "this is mostly because both clustering..." - but feature detection was evaluated together with clustering, so a conclusion specifically about clustering is unwarranted. This is a general point - can the proposed software/modular framework evaluate feature extraction separately from clustering? If yes, please demonstrate.

The reviewer is right about the fact that feature extraction was evaluated together with clustering, however, we want to stress that it is exactly the same method used by all the clustering algorithms developed in the paper. Thus, even if the feature detection method might not be optimal, it is not introducing any biases. Currently, almost all spike sorting algorithms these days are using PCA (or truncated SVD) as a feature detection method, on nearby channels where peaks are detected, and this is why we decided to only focus on this method.

(35) Lines 358-374: If SpyKING-CIRCUS 2 and TriDesClous do not provide any advantage, why include them in the MS? Many other sorters could be included as well. In other words, what do we LEARN from the failure of these sorters to compete with KS4 and Lupin? If nothing, please remove them. If something, please state the conclusions clearly.

Both these software have advantages, but we decided to keep them as they offer direct illustrations than implementation of fully integrated pipelines, other than Lupin, are possible within the proposed framework. TriDesClous2 is fast, and SpyKingCircus2 (in line with Spyking Circus [11]) is mostly tailored for *in-vitro* data, and thus can scale for more than thousands of channels while some other algorithms might not [9]. Of course, listing all the pros and cons of each software would be impossible within the scope of the paper, but we decided to keep them for illustrative purpose.

(36) Line 369: "the main advantages of these sorters lie in their modularity" - modularity is an advantage only if it is useful for something - if the sorters are modular but perform poorly, the point is nullified.

We agree with the reviewer, but we would like to point out that here, none of the modular sorters shown in the paper are performing poorly, and all have pros and cons as said in the previous point.

We believe that this is important to show that modularity can offer some options both for users and developers with respect to probe geometries, animal species, data types, ...

(37) Line 371: "on our dataset" - this is a very important limitation. See general comments, and discuss in the Limitations section of the Discussion.

We extended the paper by adding more datasets, for different probe layouts, and also real datasets with meta comparison of state of the art spike sorters (see Supplementary Figure S1).

(38) Figure 7C: the difference between the cyan (KS-like) and the orange (KS4) lines makes the KS-like irrelevant. Please improve or remove.

The reviewer is right, and we removed the KS-like pipeline, since it at the stage of the manuscript it is not yet exactly like KiloSort 4.

(39) Line 379: "each individual algorithmic steps" - should be "step".

This has been corrected

(40) Line 380: Is Lupin available for download and usage as a separate, standalone package - i.e., not as part of the spike interface framework? If not, please make it available. And if yes, indicate this clearly.

Lupin is part of the SpikeInterface project, and thus can not be installed in a standalone mode, outside of the SpikeInterface framework

(41) Line 385: "our... gigantic...effort" - avoid superlatives, especially to self.

This has been removed

References

(1) A. P. Buccino and G. T. Einevoll. Mearec: a fast and customizable testbench simulator for ground-truth extracellular spiking activity. Neuroinformatics, pages 1–20, 2020.

(2) A. P. Buccino, C. L. Hurwitz, S. Garcia, J. Magland, J. H. Siegle, R. Hurwitz, and M. H. Hennig. Spikeinterface, a unified framework for spike sorting. Elife, 9:e61834, 2020.

(3) J. M. J. Fabre, E. H. v. Beest, A. J. Peters, M. Carandini, and K. D. Harris. Bombcell: automated curation and cell classification of spike-sorted electrophysiology data.

(4) S. Garcia, A. P. Buccino, and P. Yger. How do spike collisions affect spike sorting performance? Eneuro, 9(5), 2022.

(5) S. Garcia, C. Windolf, J. Boussard, B. Dichter, A. P. Buccino, and P. Yger. A Modular Implementation to Handle and Benchmark Drift Correction for High-Density Extracellular Recordings. eNeuro, 11(2):ENEURO.0229–23.2023, Feb. 2024.

(6) A. Jain, R. Greene, C. Halcrow, J. A. Swann, A. Kleinjohann, F. Spurio, S. Graff, A. Pan-Vazquez, B. Kampa, J. Gall, S. Grün, O. Winter, A. Buccino, M. H. Hennig, and S. Musall. UnitRefine: A community toolbox for automated spike sorting curation.

(7) S. Koukuntla, T. DeWeese, A. Cheng, R. Mildren, A. Lawrence, A. R. Graves, K. E. Cullen, J. Colonell, T. D. Harris, and A. S. Charles. SLAy-ing oversplitting errors in high-density electrophysiology spike sorting. bioRxiv: The Preprint Server for Biology, page 2025.06.20.660590, 2025.

(8) J. Magland, J. J. Jun, E. Lovero, A. J. Morley, C. L. Hurwitz, A. P. Buccino, S. Garcia, and A. H. Barnett. Spikeforest, reproducible web-facing ground-truth validation of automated neural spike sorters. Elife, 9:e55167, 2020.

(9) M. Pachitariu, S. Sridhar, J. Pennington, and C. Stringer. Spike sorting with Kilosort4. Nature Methods, 21(5):914–921, May 2024. Publisher: Nature Publishing Group.

(10) C. Windolf, H. Yu, A. C. Paulk, D. Meszéna, W. Muñoz, J. Boussard, R. Hardstone, I. Caprara, M. Jamali, Y. Kfir, D. Xu, J. E. Chung, K. K. Sellers, Z. Ye, J. Shaker, A. Lebedeva, R. T. Raghavan, E. Trautmann, M. Melin, J. Couto, S. Garcia, B. Coughlin, M. Elmaleh, D. Christianson, J. D. W. Greenlee, C. Horváth, R. Fiáth, I. Ulbert, M. A. Long, J. A. Movshon, M. N. Shadlen, M. M. Churchland, A. K. Churchland, N. A. Steinmetz, E. F. Chang, J. S. Schweitzer, Z. M. Williams, S. S. Cash, L. Paninski, and E. Varol. DREDge: robust motion correction for high-density extracellular recordings across species. Nature Methods, 22(4):788–800, Apr. 2025.

(11) P. Yger, G. L. Spampinato, E. Esposito, B. Lefebvre, S. Deny, C. Gardella, M. Stimberg, F. Jetter, G. Zeck, S. Picaud, et al. A spike sorting toolbox for up to thousands of electrodes validated with ground truth recordings in vitro and in vivo. Elife, 7:e34518, 2018.
